# Insights into the Allergenic Potential of the Edible Yellow Mealworm (*Tenebrio molitor*)

**DOI:** 10.3390/foods8100515

**Published:** 2019-10-18

**Authors:** Annick Barre, Carole Pichereaux, Esmeralda Velazquez, Agathe Maudouit, Mathias Simplicien, Lorna Garnier, Françoise Bienvenu, Jacques Bienvenu, Odile Burlet-Schiltz, Cédric Auriol, Hervé Benoist, Pierre Rougé

**Affiliations:** 1 UMR 152 Pharmacochimie et Biologie pour le Développement, Université Paul Sabatier, Institut de Recherche et Développement, Faculté de Pharmacie, 35 Chemin des Maraîchers, 31062 Toulouse, France; annick.barre@univ-tlse3.fr (A.B.); esmeralda.velazquez@univ-tlse3.fr (E.V.); agathe.maudouit@gmail.com (A.M.); simplicien.mathias@gmail.com (M.S.); herve.benoist@ird.fr (H.B.); 2 Institut de Pharmacologie et Biologie Structurale, IPBS, Université de Toulouse, CNRS, UPS, 31077 Toulouse, France; Carole.Pichereaux@ipbs.fr (C.P.); Odile.Schiltz@ipbs.fr (O.B.-S.); 3 Laboratoire d’Immunologie, Hospices Civils de Lyon, Centre Hospitalier Lyon Sud, 165 Chemin du Grand Revoyet, 69495 Pierre-Bénite, France; lorna.garnier@chu-lyon.fr (L.G.); francoise.bienvenu@chu-lyon.fr (F.B.); jacques.bienvenu@chu-lyon.fr (J.B.); 4 Micronutris, 6 Rue de Partanaïs, 31650 Saint-Orens-de-Gameville, France; contact@micronutris.fr

**Keywords:** allergen, allergenicity, immunoglobulin E cross-reacting allergens, yellow mealworm, *Tenebrio molitor*, arthropods, food allergy, in vitro degranulation test, mass spectrometry

## Abstract

The edible yellow mealworm (*Tenebrio molitor*), contains an extremely diverse panel of soluble proteins, including proteins with structural functions such as muscle proteins, as well as proteins involved in metabolic functions such as enzymes. Most of these proteins display a more or less pronounced allergenic character toward previously sensitized people, especially people allergic to shrimps and other shellfish. A mass spectrometry approach following the separation of a mealworm protein, extracted by sodiumdodecyl sulfate-polyacrylamide gel electrophoresis, allowed us to identify up to 106 distinct protein fractions including molecules with structural and functional functions, susceptible to developing an allergenic potential due to the possibility of immunoglobulin E-binding cross-reactions with their counterparts occurring in shellfish. In this respect, most of the sera from people allergic to shrimps reacted with the mealworm protein extract in Western blot experiments. Moreover, the potential mealworm allergens triggered the in vitro degranulation of rat leukemic basophils transfected with the human high-affinity IgE receptor (FcεRI), upon sensitization by the IgE-containing sera from people allergic to shrimps and other shellfish foods. Owing to the large repertoire of IgE-binding cross-reacting allergens the yellow mealworm shares with other phylogenetically-related groups of arthropods, it would seem prudent to inform the consumers, especially those allergic to shellfish, by appropriate labeling on edible mealworm packages about the potential risk of developing an allergic reaction.

## 1. Introduction

Promoting the use of alternative sources of proteins to cover the food needs of the steadily increasing world population has now become a major concern for the future. Additionally, new sources of proteins are required to be produced in accordance with the energetic and environmental constraints, e.g., with an environmentally accepable carbon footprint [1,2,3,4,5,6,7,8]. In this respect, the industrial farming of edible insects and the production of insect proteins has reached a sufficient degree of improvement to meet most of these environmental requirements [9,10,11]. Beyond their nutritional value, insect proteins produced from industrial farming meet the toxicological and microbiological safety requirements allowing them to be available as safe food products [12,13,14,15,16]. However, due to the phylogenetic relationship between insects and other arthropods, there is some allergological concern since an allergic reaction may occur in consumers suffering from a shrimp allergy [17,18,19,20,21,22,23]. In agreement, hypersensitivity reactions due to the consumption of edible insects have been reported, especially in people living in countries where edible insects are traditionally consumed for a long time [24,25,26,27,28,29,30,31]. In addition, tropomyosin and arginine-kinase have been identified as the major allergens of insects [32,33,34,35,36]. More recent investigations have pointed out the risk of allergic reactions associated with the consumption of yellow mealworm (*Tenebrio molitor*), a commonly farmed edible insect in European countries, for people allergic to shrimps [37,38,39]. Different IgE-binding cross-reacting allergens including tropomyosin, α-amylase, arginine kinase, and hexamerin, have been identified as the major offending food allergens, especially of the yellow mealworm [40,41,42]. However, owing to the extreme complexity of the insect protein content, the list of IgE-binding cross-reacting allergens occurring in mealworm most probably remains incomplete and the present work was aimed at providing a more exhaustive list of potential immunoglobulin E (IgE)-binding cross-reacting allergens susceptible to occur in mealworm.

## 2. Experimental Procedure

### 2.1. Yellow Mealworm Protein Extract

A total of 150 mg of yellow mealworm flour (Micronutris) was suspended in 850 µL of tris-buffered saline (Tris 20 mM, NaCl 130 mM, ethylenediaminetetraacetic acid 2 mM, pH 7.4) and homogenized by two grinding steps of 40 s each with steel pellets in a Fast Prep-24 homogenizer (MP Biomedicals, Illkirch, France). The resulting slurry was centrifuged at 13,000 rpm for 10 min at 4 °C. The supernatant fraction was carefully collected while avoiding the floating lipid layer and stored at −20 °C until used. The protein content in the mealworm protein extract was estimated by the bicinchoninic method kit (Pierce) [43], using bovine serum albumin (Sigma, Saint Quentin Fallavier, France) as a standard.

### 2.2. Patient Sera

Well documented blood samples with various specific IgE content were drawn after receiving the informed consent of 21 patients experiencing shrimp anaphylaxis. Their reactivity towards the yellow mealworm extract was checked in dot-plot experiments and Western blot analyses. Dust mite allergic patient sera (*n* = 13) were also checked for IgE-reactivity towards the mealworm extract. A new table indicating for 21 sera from shrimp-allergic patients and 13 sera from dust mite allergic patients (Table A1), the measured specific IgE contents (express as kU·L^−1^) and cross-reactivities, is proposed as an Annex to the text.

### 2.3. Dot-Blot Screening

The shrimp-allergic patient sera were screened for their IgE-reactivity towards the yellow mealworm protein extract using a dot-blot technique. The mealworm protein extract (1 µL) was spotted on a nitrocellulose membrane at increasing protein concentrations (1, 5, 10, 15, and 20 μg·mL^−1^), and checked for IgE-binding reactivity with the shrimp-allergic patient sera using goat horseradish peroxydase (HRP)-labelled antihuman IgE (Invitrogen) as a probe, as described in §4.3 for Western blot experiments. Dust mite allergic patient sera (*n* = 13) were also checked for IgE-reactivity towards the mealworm extract but only two of them gave a readily positive result in dot-blot experiments.

### 2.4. SDS-PAGE and Western Blot Experiments

SDS-PAGE was performed in 12.5% acrylamide gels and protein fractions were stained with Coomassie blue R250 (BioRad, Marnes-la-Coquette, France) [44]. Western blot experiments were performed after a semi-dry transfer of the protein fractions separated by SDS-PAGE on nitrocellulose sheets (Amersham, Les Ulis, France). After an overnnight incubation in 10 mM PBS (pH 7.4) containing 5% (*v*/*v*) skimmed milk, membranes were soaked in properly 1:10 diluted shrimp-allergic patient sera and incubated in a moist chamber for 2 h at room temperature. After three washings of 10 min each with the same buffer, membranes were soaked and incubated for 1 h under gentle stirring, in goat HRP-labelled antihuman IgE (Invitrogen) diluted 1:1500. Following three washings of 10 min each with buffer, the immunolabelled protein fractions were revealed using the SuperSignal West Dura substrate (ThermoScientific, Illkirch, France) by chemiluminescence.

Bi-dimensional SDS-PAGE was performed using strips with pH 3.0–10.0 (ZOOM Strip pH 3-10NL-Novex, Illikirch, France). The yellow mealworm protein extract was mixed with the rehydration solution BioRad (CHAPS 4%, 0.2% BioLyte 1X, urea 8M, DTT 20 mM), added to a final volume of 155 mL/strip, and strips were rehydrated overnight in the scelled ZOOM IPGRunner cassettes (Thermo Fisher Scientific, Illkirch, France). The IEF migration was conducted consecutively at 175 V (15 min), up to 2000 V (45 min), and at 2000 V (45 min). Strips were incubated for 15 min in the reducing solution (625 μL 4X NuPage LDS Sample Buffer, 2075 μL dist. water, 300 μL NuPage Sample Reducing Agent 10X), and further incubated in the alkylation solution (300 μL 4X NuPage LDS Sample Buffer, 2700 μL dist. water, 70 mg iodoacetamide) for an additional 15 min. Strips were then introduced at the top of a 12.5% acrylamide SDS-PAGE gel, covered with 0.5% (*w*/*v*) molten agarose, and run at 200 V/30 mA using a Tris-glycine running buffer (pH 8.3). Protein spots were stained with Coomassie blue R250 (BioRad, Marnes-la-Coquette, France).

### 2.5. Degranulation Test

Degranulation of rat leukemic cells RBL SX38 [45], transfected with the high-affinity human FcεRI receptor, in the presence of the meaworm protein extract, was performed in 96-well plates on RBL SX38 cells (2.10^5^ cells) cultured in minimum essential medium (MEM) Gibco (Thermo Fisher Scientific, Illkirch, France) containing 10% (*v*/*v*) foetal calf serum, for 2 days at 37 °C. Cells were then cultured in the MEM medium containing 2% (*v*/*v*) sample sera from different shrimp-allergic patients (diluted 1:40, *v*/*v*), for 2 days at 37 °C. Cells were washed two times with tyrode buffer (Sigma, Saint-Quentin Fallavier, France and incubated for 45 min at 37 °C in tyrode/D2O (1:1, *v*/*v*) buffer containing 1.0 μL of mealworm protein extract. Cells were further incubated for 90 min at 37 °C in 50 μL of 0.05 M citrate buffer (pH 4.5) containing 1.3 mg/mL of *p*-nitrophenyl N-acetyl-β-D-glucosaminide (Sigma, Saint-Quentin Fallavier, France) as a chromogenic substrate for the hexosaminidase. The color reaction was stopped by adding 100 μL of 0.4 M glycine buffer (pH 10.7) and the absorbance was spectrophotometrically measured at 410 nm. Degranulation obtained under similar conditions by probing a shrimp protein extract, was used as a sample for the 100% degranulation value. 

### 2.6. Digestion and Nano-LC-MS/MS Analysis

Proteome samples were reduced and alkylated using equilibration buffers containing dithiothreitol and iodoacetamide, and loaded onto a 12% SDS-polyacrylamide gel. After Instant Blue (Invitrogen) staining of the gel, bands were excised and digested by the addition of 60 µL of a solution of modified trypsin in 25 mM NH_4_HCO_3_ (10 ng/µL, sequence grade, Promega, Charbonnières, France). The mixture was incubated at 37 °C overnight. The peptides mixtures were analyzed by nano-LC-MS/MS (Liquid chromatography–mass spectrometry/Mass spectrometry) using nanoRS UHPLC system (a nanoliter-range ultra high performance liquid chromatography, Dionex, Amsterdam, The Netherlands) coupled to an LTQ-Orbitrap Velos mass spectrometer (Thermo Fisher Scientific, Bremen, Germany). Five microliters of sample were loaded on a C18 precolumn (300 µm inner diameter × 5 mm, Dionex) at 20 µL/min in 5% acetonitrile, 0.05% trifluoroacetic acid. After 5 min desalting, the precolumn was switched in line with the analytical C18 column (75 µm inner diameter × 15 cm; in-house packed) equilibrated in 95% solvent A (5% acetonitrile, 0.2% formic acid) and 5% solvent B (80% acetonitrile, 0.2% formic acid). Peptides were eluted using a 5%–50% gradient of solvent B during 105 min at a 300 nL min^−1^ flow rate. The LTQ-Orbitrap was operated in data-dependent acquisition mode with the Xcalibur software. Survey scan MS spectra were acquired in the Orbitrap on the 300–2000 *m*/*z* range with the resolution set to a value of 60,000. The 20 most intense ions per survey scan were selected for collision-induced dissociation (CID) fragmentation, and the resulting fragments were analyzed in the linear trap (LTQ). Dynamic exclusion was used within 60 s to prevent repetitive selection of the same peptide.

The Mascot Daemon software (version 2.5, Matrix Science, London, UK) was used to perform database searches in batch mode with all the raw files acquired on each sample. To automatically extract peak lists from Xcalibur raw files, the Extract_msn.exe macro provided with Xcalibur (version 2.2 SP1.48, Thermo Fisher Scientific, Illkirch, France) was used through the Mascot Daemon interface. The following parameters were set for creation of the peak lists: parent ions in the mass range 400–4500, no grouping of MS/MS scans, and threshold at 1000. A peak list was created for each analyzed fraction (i.e., gel slice), and individual Mascot searches were performed for each fraction. Data were searched against all entries in the Tenebrionidae 20170606 protein database (22,376 sequences; 10,097,372 residues). Oxidation of methionine and carbamidomethylation of cysteine were set as variable modifications for all Mascot searches. Specificity of trypsin digestion was set for cleavage after Lys or Arg except before Pro, and one missed trypsin cleavage site was allowed. The mass tolerances in MS and MS/MS were set to 5 ppm and 0.8 Da, respectively, and the instrument setting was specified as “ESI-Trap”. Mascot results were parsed and validated with an in-house software called Proline (ProFi Proteomics, France). The target-decoy database search allowed us to control and estimate the false positive identification rate of our study, and the final catalogue of proteins presented an estimated false discovery rate (FDR) below 1% for peptides and proteins.

### 2.7. Bioinformatics

Multiple amino acid sequence alignments were carried out with CLUSTAL-X [46] using the stuctural Risler’s matrix for homologous residues [47]. 

Except for the three-dimensional structures of apoL-III of *Locusta migratoria* (protein data bank (PDB) code 1AEP [48] and 1LS4 [49]) and *Manduca sexta* (PDB code 1EQ1) [50], and the 12 kDa hemolyph protein of *Tenebrio molitor* (PDB code 1C3Z) [51], which are available at the Protein Data Bank (PDB), the three-dimensional models of other apoL-III, 12 kDa HLP (hemolymph protein) and larval cuticle proteins were built by homology modeling with the YASARA Structure program [52]. The three-dimensional structure of the *Locusta migratoria* apoL-III (PDB code 1AEP) was used as a template to build up to 3 different models for each of the modelled apoL-IIIs. Finally, hybrid models of the apoL-III of *Acheta domesticus* (house cricket), *Bombyx mori* (silkworm), *Galleria mellonella* (greater wax moth), *Musca domestica* (housefly), and *Schistocerca americana* (American grasshopper) were built up from the different previous models. Similarly, the three-dimensional structures of odorant binding proteins (OBP) from *Apis mellifera* (PDB code 3S0D) [53], *Anopheles gambiae* (PDB code 3R1P) [54], AtraPBP1 from *Amyelois transtyella* (PDB code 4INW) [55], *Antheraea polyphemus* PBP1 (PDB code 2JPO) [56], *Bombyx mori* GOBP2 (PDB code 2WCJ) [57], chemosensory protein from *Mamestra brassicae* (PDB code 1N8V) [58], and chemosensory protein 1 from *Bombyx mori* (PDB code 2JNT) [59]), were used as templates for the building of the 12 kDa HLP models. Finally, hybrid models of the 12 kDa HLP (hemolymph protein) of *Manduca sexta* (Carolina sphinx moth), *Rhynchophorus ferrugineus* (red palm weevil), and *Tenebrio molitor* (yellow mealworm) were built up from the different previous models. A single protein template, the crystal structure of p53 epitope-scaffold of a cysteine protease in complex with human MDM2 protein (5SWK), available at the PDB, was used for building the 3D-model for the larval cuticle proteins (LCP) of *Tenebrio molitor*, *Bombyx mori*, *Locusta migratoria*, *Musca domestica,* and *Tribolium castaneum* (red flour beetle). PROCHECK [60], Atomic Non-Local Environment Assessment (ANOLEA) [61], and the calculated QMEAN scores [62,63], were used to assess the geometric and thermodynamic qualities of the three-dimensional models. Using ANOLEA to evaluate the models, only a few residues of the different models exhibited an energy over the threshold value. Both residues are mainly located in the loop regions connecting the α-helices or β-sheets in the models. The calculated QMEAN6 score of all of the models gave values > 0.5. 

The ConSurf server was used to discriminate between conserved and variable regions in the allergen models [64]. Molecular cartoons were drawn with YASARA [52] and Chimera [65].

## 3. Results

### 3.1. Dot-Blot Screening of Shrimp-Allergic Patient Sera Towards a Yellow Mealworm Protein Extract

**Figure 1:**
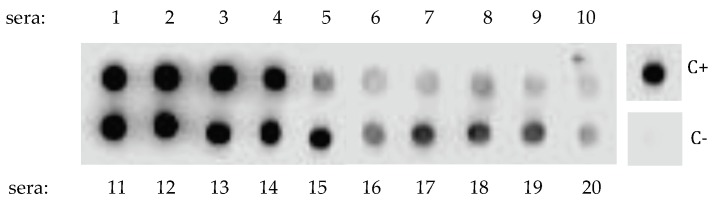
Dot-blot screening of the yellow mealworm protein extract (protein concentration of 15 mg mL^−1^) by 20 sera from shrimp-allergic patients, revealed with an anti-IgE second antibody. Data not shown for the single nonreactive serum #21. Depending on the patient sera, the IgE cross-reacting antibodies interacted strongly (dark dot) or weakly (grey dot) with the immobilized protein extract. No data for sera from dust mite allergic patients were shown because most of them (11/13), gave negative results. C+ and C− correspond to the positive and negative controls (first IgE antibody omitted) performed with a shrimp protein extract, respectively.

A dot-blot screening of a number of sera from patients allergic to the shrimp (*n* = 21) and the dust mite *Dermatophagoides peteronyssinus* (*n* = 13) toward a mealworm protein extract immobilized on nitrocellulose sheet resulted in positive responses for almost all (20/21) of the checked shrimp sera (Figure 1), whereas a very few dust mite sera reacted positively (2/13). However, depending on the checked shrimp sera, large discrepancies with respect to the IgE-binding capacity were noticed between strongly and more weakly IgE-reacting serum samples. 

### 3.2. Identification of IgE-Binding Cross-Reactive Allergens in the Yellow Mealworm Protein Extract

**Figure 2:**
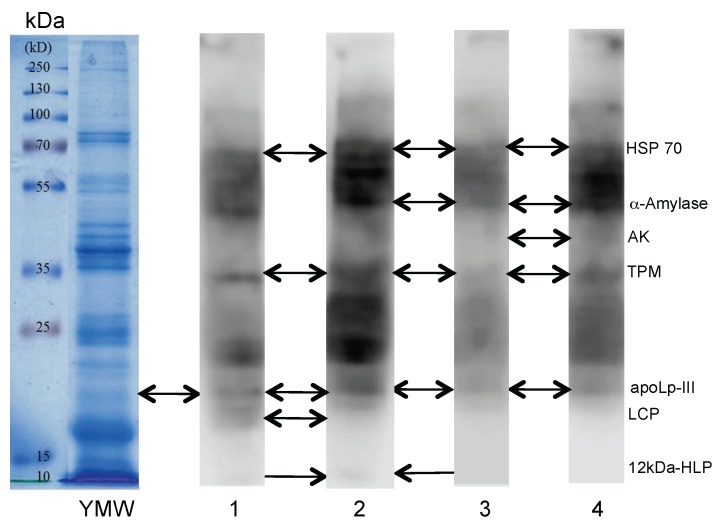
SDS-PAGE of the yellow mealworm (second line labelled, YMW). The first line corresponds to the electrophoretic migration of the MW markers (kDa). Lines 1, 2, 3, and 4 correspond to the Western blots of a yellow mealworm extract revealed by IgE-containing sera from different patients allergic to shrimp. The identified allergenic fractions HSP (heat shock protein) 70, α-amylase, arginine kinase (AK), tropomyosin (TPM), apolipophorin-III (apoLp-III), larval cuticle protein (LCP), and 12 kDa hemolymph protein (12kDa-HLP (hemolymph protein)) of mealworm are indicated by arrows.

Some of the IgE-binding protein fractions reavealed in Western blot experiments using different sera from patients allergic to shrimp corresponded to allergens previously identified in yellow mealworm protein extracts, e.g., tropomysosin, HSP (heat shock protein) 70, α-amylase, and arginine kinase (Figure 2) [36]. Many other mealworm allergens also interacted with the cross-reacting IgE-binding sera but have not been clearly identified by mass spectrometry. Other allergen proteins with low molecular weights have been identified at the bottom of the SDS-PAGE gels. They consist of predominantly hydrophobic proteins that are involved in the transportation of hydrophobic ligands like the apolipophorin-III (apoLp-III), often associated to the larval cuticle protein (LCP), which is abundantly represented in the yellow mealworm, and to a weaker IgE-binding signal corresponding to the 12 kDa hemolymph protein (12 kDa-HLP). 

**Figure 3:**
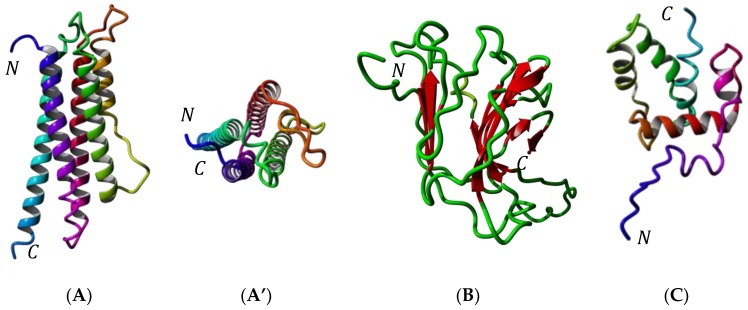
(**A**) Ribbon diagram of apolipophorin-III of *Tenebrio molitor* (sagital view). Alpha-helices numbered α1–α5 are differently colored. *N* and *C* correspond to the *N*- and *C*-terminus of the polypeptide chain, respectively. (**A’**) Ribbon diagram of apolipophorin-III of *T. molitor*: upper view showing the arrangemernt of α-helices around a central hydrophobic pocket. (**B**) Ribbon diagram of the larval cuticlar protein (LCP) of *T. molitor*, built up from the association of two antiparallel bundles of β-sheet in a β-sandwich structure. (**C**) Ribbon diagram of the 12 kDa hemolymph protein (12 kDa HLP) of *T. molitor*, built up from the association of six α-helices around a central hydrophobic pocket. Alpha-helices are differently colored.

**Figure 4:**
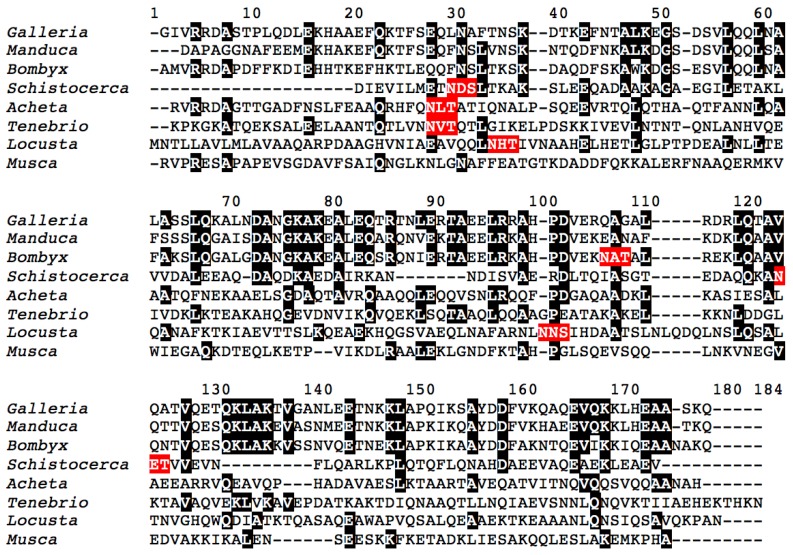
Multiple alignment of the apolipophorin-III protein from the edible insects Galleria mellonella, Manduca sexta, Bombyx mori, Schistocerca gregaria, Acheta domesticus, Tenebrio molitor, Locusta migratoria, and Musca domestica. Identical amino acid residues are in black boxed white letters. Putative N-glycosylation sites are colored red.

**Figure 5:**
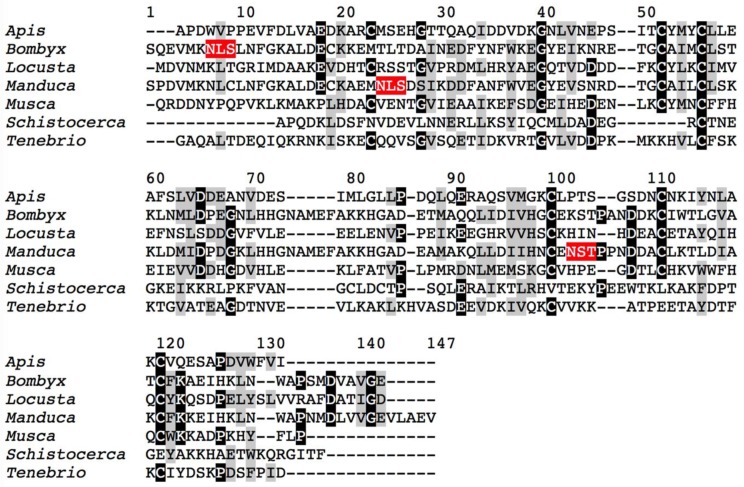
Multiple alignment of 12 kDa HLP and OBP (odorant bindin proteins) from the edible insects *Apis mellifera*, *Bombyx mori, Locusta migratoria, Manduca sexta, Musca domestica, Schistocerca gregaria,* and *Tenebrio molitor*. Identical amino acids residues are in black boxed white letters whereas structurally homologous residues are grey boxed. Putative *N*-glycosylation sites are colored red.

**Figure 6:**
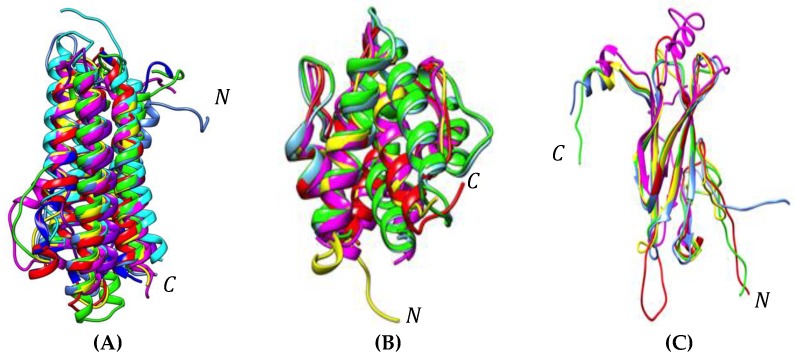
(**A**) Superposition of the differently colored three-dimensional models of apoLP-III of the edible insects *Acheta domesticus* (blue)*, Bombyx mori* (green)*, Galleria mellonella* (magenta)*, Locusta migratoria* (yellow)*, Manduca sexta* (deep blue)*, Musca domestica* (cyan)*, Schistocerca gregaria* (purple), and *Tenebrio molitor* (red). (**B**) Superposition of the differently colored three-dimensional models of 12 kDa HLP and OBP of the edible insects *Tenebrio molitor* (red), *Bombyx mori* (blue), *Manduca sexta* (green), *Onchorhynchus ferrugineus* (magenta), and *Apis mellifera* (yellow). (**C**) Superposition of the differently colored three-dimensional models of cuticle protein of the edible insects *Tenebrio molitor* (red), *Bombyx mori* (green), *Locusta migratoria* (magenta), *Musa domestica* (yellow), and *Tribolium castaneum* (blue). *N* and *C* indicate the *N*- and *C*-terminal ends of the polypeptide chains.

Molecular modeling of these allergenic proteins (Figure 3) reveals the occurrence of hydrophobic cavities most probably adapted to the binding of hydrophobic ligands like fatty acids (apoLp-III) [48,49,50] or pheromone-like ligands (12 kDa-HLP) [51,52,53,54,55,56,57,58,59]. These newly identified insect allergens are reminscent of other proteins like the lipid transfer protein (LTP) allergens occurring in pollens and fruits, which also participate in the transport of hydrophobic compounds such as Pathogenesis-Related PR-14 members of the plant defense protein [66]. All of the newly identified insect allergens consist of ubiquitous proteins that share very conserved three-dimensional structures but rather poorly conserved amino acid sequences among the phylogenetically related insect species (Figure 4 and Figure 5). In this respect, all of these ubiquitous allergens of different origins exhibit similar three-dimensional structures that are readily superposable even though their amino acid sequences are much less conserved (Figure 6). 

**Figure 7:**
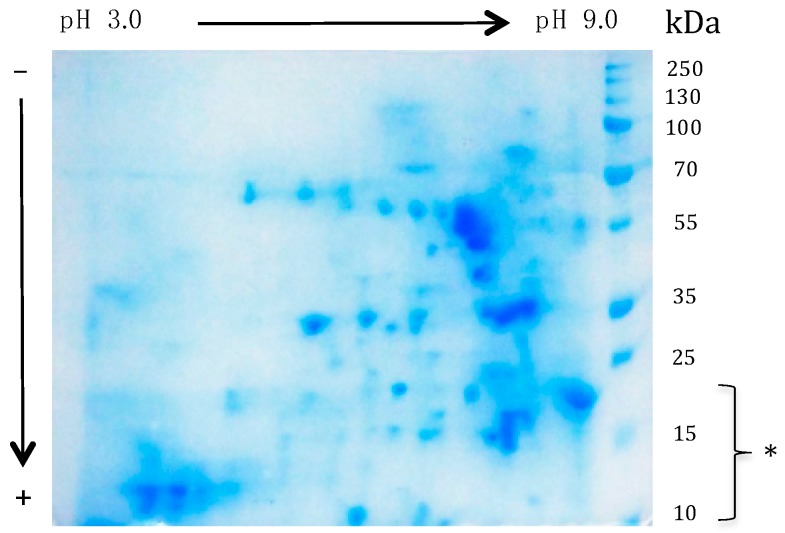
Bidimensional SDS-PAGE electrophoregram of the mealworm protein extract stained with Coomassie blue. A number of protein fractions with a MW in the range between 10 kDa and 100 kDa, occur between pH 3.0 and pH 9.0. Low MW protein fractions containing the apoLp-III, LCP and the 12 kDa-HLP allergens occur at the bottom of the gel (*).

However, SDS-PAGE of the mealworm protein extract does not account for the extreme diversity in the soluble protein content of the mealworm. In this respect, the Coomassie blue-stained 2D SDS-PAGE gels offer a more relevant picture of the protein diversity occurring in the mealworm protein extract, which exhibits a number of protein spots occurring in the range between pH 3.0 and pH 9.0, with MW ranging from 10 kDa up to 100 kDa (Figure 7). This apparent diversity of the soluble protein content suggests that a huge number of soluble protein fractions occurring in the mealworm protein extract could behave as potential cross-recting IgE-binding allergens.

### 3.3. Characterization of the Potential Allergenic Protein Repertoire of the Mealworm Protein Extract

A more sophisticated SDS-PAGE coupled to a mass spectrometry characterization allowed the identification of up to 106 distinct protein fractions in the mealworm protein extract (Table 1). 

**Figure 8:**
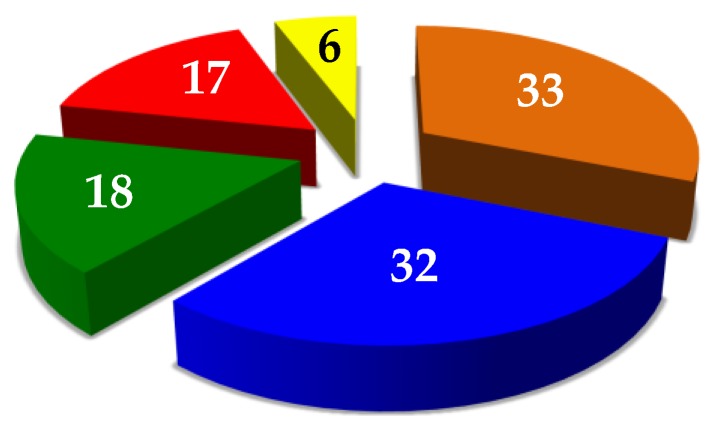
Distribution of the 106 proteins identified in the *T. molitor* extract, according to their potential functions: proteins with a functional role (33, orange), enzymes (32, blue), proteins with a structural function (18, green), muscle proteins (17, red), and proteins with other roles (6, yellow).

The identified proteins may be arbitrarily distributed in different categories according to their structure–function relationships, such as functional proteins (33 total, 31%), enzymes (32 total, 30%), structural proteins (18 total, 17%), muscle proteins (17 total, 16%), and others (6 total, 6%) (Figure 8).

Most of the protein fractions belonging to the different structural and functional categories consist of cross-reacting IgE-binding allergens that have been previously identified in various insects and other groups of arthropods (dust mites, shrimps) and mollusks (mussels, oysters, snails) [67,68,69,70,71,72,73,74,75,76]. In this respect, a number of proteins involved in muscle contraction (actin, myosin) and others associated with muscle contraction (tropomyosin, troponin, actinin) occupy a large part of the allergen list. Allergens with enzymatic functions are also highly represented. It is noteworthy that the *T. molitor* list includes most of the allergens identified so far in both the American (*Periplanta americana*) and European (*Blattella germanica*) cockroaches. 

### 3.4. The Cross-Reacting Allergens of the Mealworm Protein Extract Are Functional in Degranulation Tests In Vitro

**Figure 9:**
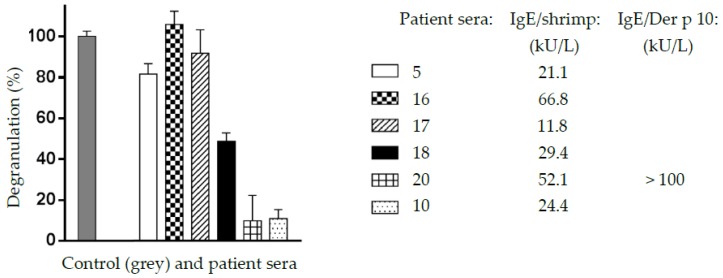
Degranulation potency, expressed as degranulation percentage (%), caused by the mealworm protein extract, using six different sera (5,16,17,18,20,10) from shrimp-allergic patients as IgE-binding cross-reacting allergens toward the mealworm allergenic proteins. A shrimp protein extract (grey histogram) was used as the 100% degranulation value (control C). Each value is the mean of six separate measurements. Values for specific IgE in sera 5, 16, 17, 18, 20, and 10, are indicated.

In vitro degranulation experiments performed with different cross-reacting sera from shrimp-allergic patients as probes resulted in positive results for all of the assayed sera (Figure 9). However, depending on the shrimp patient sera, a large discrepancy was observed in the degranulation potency of the mealworm protein extract. These results suggest that the cross-reacting IgE-binding allergens of the mealworm protein extract are apparently functional and might trigger some allergenic response in previously sensitized people, e.g., in shrimp-allergic people. These degranulation data are in agreement with those previously reported for *T. molitor* [36,37].

## 4. Discussion

A few protein members in the mealworm protein extract were identified as new potential allergens, including the apolipophorin-III (apoL-III), the larval cuticular protein (LCP), and the 12 kDa hemolymph protein (12 kDa HLP). Both apolipophorin-III and 12 kDa HLP proteins participate in the binding and transport of hydrophobic ligands like fatty acids and derivatives for apoL-III and LCP or 12 kDa HLP [48,49,50,51,52,53,54,55,56,57,58,59]. The 12 kDa HLP consist of ubiquitous proteins closely related to the odorant binding proteins OBP widely distributed in insects, exhibiting conserved three-dimensional structures (Figure 6) even though their amino acid sequences are moderately conserved (Figure 5). However, the nature of small hydrophobic ligands they transport still remains a matter of debate. The larval cuticle proteins LCP represent a rather well conserved group of proteins possessing a chitin-binding domain. They play a key role in the deposition of the newly synthesized chitin chains forming the chitinous shell during the ecdysone-driven molt periods of insect larvae. They closely resemble some other pupal cuticle proteins, which also consist of a large hydrophilic central part with a β-fold flanked by two hydrophobic regions containing alanin-rich repetitive motifs [77].

Most, if not all, of the protein allergens of edible insects identified so far consist of ubiquitous proteins widely distributed in other arthropods including dust mites, crustaceans and other insects, mollusks, and nematods as well [78]. However, the number of proteins identified as insect allergens still remains very scarce compared to the diversity and complexity of their protein content. In this respect, some of the insect protein components, e.g., the hemolymph proteins for all the insect groups and muscle proteins for the flying insects, display an extreme diversity that reflects the successful adaptation of this group of animals to diverse ecological conditions during their evolution. Accordingly, it is expected that the still poorly understood allergen repertoire of edible insects will become progressively enriched by new proteins with allergenic properties. In fact, the difficulties in identifying and obtaining clinically well documented sera from insect allergic patients is a major breakthrough in the identification of new insect allergens. Even in countries where edible insects have traditionally been a significant part of the diet, only a few reports are available on the food allergic reactions attributable to eating crude or roasted insects [26]. In future years, the forthcoming introduction of insect flour as a protein-enriched supplement in the diet, will problably result in an increase of allergic manifestations among consumers. It will thus be possible to identify new allergenic proteins and, eventually, allergens specifically associated to some edible insect species.

Beside their potential negative impact on people suffering from shrimp allergy, mealworm protein flour should be used as a protein-rich source to supplement the diet for the treatment and care of severely undernourished people, owing to its excellent nutritional properties [79,80,81,82]. In addition, proteins from the yellow mealworm protein extract might bring some beneficial effect in mild to moderate hypertensive consumers, by providing angiotensin I-converting enzyme (ACE) inhibitory peptides upon digestive proteolysis. Like many other inhibitory peptides previously identified in the edible insect protein hydrolysates, ACE inhibitory peptides derived from *Tenebrio molitor* larva protein hydrolysate have been known for a long time [83,84]. Introducing some yellow mealworm protein flour into the diet should create a relevant source of ACE inhibitory peptides available for helping mildly hypertensive people to regulate their blood pressure. Yellow mealworm larvae also contain bioactive compounds with inhibitory activities for factor Xa and platelet aggregation [85]. Moreover, due to their antioxidant activities, introduction of edible insects in the diet could be beneficial for other oxidative-stress associated health disorders [86,87]. However, in addition to their beneficial properties, the sensory attributes of edible insects might be deeply investigated for improving the acceptance of edible insects in food production, especially by people of Western countries [88]. 

In spite of their beneficial properties, the incorporation of edible insects or insect proteins into the diet of shellfish allergic people still remains hazardous since the risk of an anaphylactic response can not be ruled out. In this respect, an appropriate labelling of edible insect packages and food product containing insect proteins is advisable to inform the consumers of such a potential risk. However, some recent reports demonstrate that appropriate food processing methods, e.g., thermal processing or enzymatic proteolysis, can drastically reduce the risk of cross-reactivity and allergenicity associated to edible insect proteins [37,38,89].

## Figures and Tables

**Table 1:** List of proteins identified in the yellow mealworm (*Tenebrio molitor*) protein extract. Proteins are ranked by decreasing scores. Proteins already identified as IgE-binding allergens from arthropods are in bold.

Score:	Protein:	Score:	Protein:
2984.019	**α-Myosin**	222.675	28 kDa dessication stress protein
1214.674	Aldehyde oxidase	219.963	**Masquerade-like serine protease**
1066.813	Ca-transporting ATPase	213.041	13 kDa hemolymph protein d
1025.416	**Actin 87E**	211.853	Larval cuticle protein A3A
940.811	86 kDa Early stage encapsulation	211.417	12 kDa hemolymph protein c
	inducing protein	210.607	**Myosin light chain alkali**
895.841	**Tropomyosin 2**	209.594	Fructose-biphosphate aldolase
806.565	**Actin 5C**	209.332	**Serine proteinase**
765.02	**Actinin α**	207.818	**Glutathione S-transferase**
740.12	**Tubulin β**	201.438	13 kDa hemolymph protein b
664.062	Prophenoloxidase	198.188	Vitellogenin receptor
609.664	**Tropomyosin 1**	190.799	**Troponin C**
	ATP synthase subunit β	189.968	Calreticulin
504.441	**α-Amylase**	187.099	4.5 LIM domains protein 5
493.123	**Tubulin α**	184.454	Cytochrome c2
474.845	Odorant binding protein OBP 14	179.106	Nucleoside triphosphatase A
474.053	**Apolipophorin-III**	177.997	Muscle-specific protein 20
446.015	Myosin heavy chain, non-muscle	176.202	Muscle LIM protein Mlp84B
445.547	**Arginine kinase**	171.809	Enolase
439.842	**HSP 70**	171.39	Obstractor C2
423.306	Filamin-A	168.27	Protein disulfide-isomerase
395.328	12 kDa hemolymph protein a	164.625	13 kDa hemolymph protein c
386.89	Twitchin-like protein	161.351	Phosphoglycerate kinase
370.18	**Cockroach allergen-like protein**	159.53	Chemosensory protein CSP12
363.618	Serpin 1	157.539	α-Spectrin
359.558	ATP synthase subunit α	157.443	14.3.3. ζ
340.168	**Hexamerin 2**	155.443	Glycogenin-1-like protein
338.385	Glyceraldehyde-3-phosphate	151.351	Phosphoglycerate kinase
	dehydrogenase	147.874	Ferritin
337.266	56 kDa Early stage encapsulation	142.987	Calumenin
	inducing protein	141.375	Cathepsin L11
326.698	Voltage-dependant anion-selective	140.034	Ribosomal protein S3
	channel protein	132.912	Nucleobindin-2 like protein
315.75	**Paramyosin long form**	130.118	**Trypsin**
313.141	Serpin 93 kDa	129.432	G protein-coupled receptor kinase 1
296.596	Larval cuticle protein A1A	124.835	THP isoform 84aa-XY
286.543	Serpin 40	122.826	Chitin deacetylase 1
280.057	Cathepsin F10	122.326	Myosin regulatory light chain-2
279.884	α-1,4-glucan phosphorylase	122.024	Malate dehydrogenase
278.386	Larval cuticle protein A2B	121.525	Cuticular protein
278.215	**Troponin T**	118.956	Chitinase
276.285	Melanization-related protein	116.322	Glucose-6-phosphate isomerase
273.213	**Paramyosin short form**	99.944	Carbonyl reductase 1
269.133	Peptidyl-prolyl *cis*-*trans* isomerase	98.68	Protein lethal/essential for life
260.731	**Apolipophorin-like protein**	96.268	Transketolase-like protein 2
259.533	**Myosin heavy chain, muscle**	89.762	Multiple coagulation factor
259.092	HSP 60		deficiency protein 2
254.399	V-type proton ATPase subunit β	87.931	60S ribosomal protein L31
251.439	Pyruvate kinase	87.574	ATP carrier protein
249.266	Melanin-inhibiting protein	87.104	**Lysosomal aspartic protease**
247.353	Serpin 48	78.888	Aspartate aminotransferase
246.2	**Superoxide dismutase** **(Cu-Zn)**	76.674	Elongation factor 1-α
237.263	12 kDa hemolymph protein b	69.44	Adenosylhomocysteinase
229.745	RNA-binding protein squid-like	59.528	Muscular protein 20
227.752	Sarcalumenin	34.596	Reticulon-like protein

## Appendix A

**Table A1:** Sera from patients allergic to shrimp (1a–21a) and dust mite (1b–13b), and specific IgE values.

Seum:	Gender:	Shrimp Specific IgE (kU·mL^−1^)	Der p 10 Specific IgE (kU·mL^−1^)
1a	Female	81.1	
2a	Female	45.3	
3a	Female	49.8	
4a	Male	28.2	>100
5a	Female	21.1	
6a	Male	>100	
7a	Female	66.8	
8a	Male	>100	
9a	Male	33.9	
10a	Female	24.4	
11a	Male	61.8	
12a	Female	54.8	
13a	Male	11.2	40.8
14a	Female	>100	
15a	Female	18.8	
16a	Female	66.8	
17a	Male	11.8	
18a	Male	29.4	
19a	Female	>100	
20a	Male	52.1	>100
21a	Female	10.2	
1b	Male		>100
2b	Female		>100
3b	Female		>100
4b	Female	17.0	>100
5b	Female		>100
6b	Female		>100
7b	Female		>100
8b	Male		>100
9b	Male	1.4	>100
10b	Female	27.3	>100
11b	Male	2.4	84.6
12b	Female	0.1	13.1
13b	Male		>100
